# Expression and display of UreA of *Helicobacter acinonychis *on the surface of *Bacillus subtilis *spores

**DOI:** 10.1186/1475-2859-9-2

**Published:** 2010-01-18

**Authors:** Krzysztof Hinc, Rachele Isticato, Marcin Dembek, Joanna Karczewska, Adam Iwanicki, Grażyna Peszyńska-Sularz, Maurilio De Felice, Michał Obuchowski, Ezio Ricca

**Affiliations:** 1 Department of Structural and Functional Biology, Federico II University of Naples, via Cinthia 4, Naples 80126, Italy; 2 Department of Medical Biotechnology, Intercollegiate Faculty of Biotechnology UG-MUG, Dębinki 1, Gdańsk 80-211, Poland; 3 Tri-City Animal Laboratory, Medical University of Gdańsk, Dębinki 1, Gdańsk 80-211, Poland

## Abstract

**Background:**

The bacterial endospore (spore) has recently been proposed as a new surface display system. Antigens and enzymes have been successfully exposed on the surface layers of the *Bacillus subtilis *spore, but only in a few cases the efficiency of expression and the effective surface display and have been determined. We used this heterologous expression system to produce the A subunit of the urease of the animal pathogen *Helicobater acinonychis*. Ureases are multi-subunit enzymes with a central role in the virulence of various bacterial pathogens and necessary for colonization of the gastric mucosa by the human pathogen *H. pylori*. The urease subunit UreA has been recognized as a major antigen, able to induce high levels of protection against challenge infections.

**Results:**

We expressed UreA from *H. acinonychis *on the *B. subtilis *spore coat by using three different spore coat proteins as carriers and compared the efficiency of surface expression and surface display obtained with the three carriers. A combination of western-, dot-blot and immunofluorescence microscopy allowed us to conclude that, when fused to CotB, UreA is displayed on the spore surface (ca. 1 × 10^3 ^recombinant molecules per spore), whereas when fused to CotC, although most efficiently expressed (7-15 × 10^3 ^recombinant molecules per spore) and located in the coat layer, it is not displayed on the surface. Experiments with CotG gave results similar to those with CotC, but the CotG-UreA recombinant protein appeared to be partially processed.

**Conclusion:**

UreA was efficiently expressed on the spore coat of *B. subtilis *when fused to CotB, CotC or CotG. Of these three coat proteins CotC allows the highest efficiency of expression, whereas CotB is the most appropriate for the display of heterologous proteins on the spore surface.

## Background

Surface display systems are a powerful biological tool with a variety of applications in the development of live vaccines, generation of biocatalysts or biosensors, treatment of microbial infections and screening of peptide libraries [1,2]. Several approaches to display heterologous proteins in bacteria and phages have been developed and extensively reviewed [2,3]. In general, methods to display heterologous proteins involve the construction of gene fusions that code for a chimera formed by a carrier protein that anchors a heterologous passenger protein on the cell surface [3]. Similar approaches have also been used for displaying heterologous antigens [4,5] or enzymes [6,7] on the surface of endospores (spores) of *Bacillus subtilis*. The spore surface (spore coat) is formed by over 50 proteins organized into a inner and an outer layer. Components of the outer layer, selected for their surface location [4] or relative abundance [5-7], have been used as carrier proteins.

Spores are extremely stable life forms generated by gram-positive bacteria of the *Bacillus *and *Clostridium *genera in response to harsh environmental conditions that do not allow cell growth and survival. In the spore form these bacteria can survive indefinitely in the absence of nutrients and can resist UV irradiation, extreme temperature and exposure to lytic enzymes and toxic chemicals [8]. Spore coat proteins are produced in the bigger cell (mother cell) and assembled around the forming spore in the mother cell cytoplasm, thus eliminating the need of secretion signals and the constrains due to translocation across a membrane. In addition, several coat proteins are dispensable for the formation of an apparently normal spore and, for this reason, their manipulation to incorporate the heterologous part usually does not affect spore structure [9]. With respect to systems based on the use of phages or bacterial cells, the spore-display system provides also additional advantages, such as high stability and safety due to the unusual properties of this peculiar cell form [8]. The commercial use of spores of various species of the *Bacillus *genus as probiotics or for the oral prophylaxis of gastrointestinal disorders, clearly proves the safety of spores of these species [10].

So far, two coat proteins have been used to display heterologous antigens, CotB and CotC. Both proteins are in the outermost layer of the coat, from where they can be extracted as 66 kDa (CotB) and 12 kDa (CotC) species [11,12]. CotB has been used to display the C-terminal fragment of the tetanus toxin (TTFC) [4], domains 1b-3 and 4 of the Protective Antigen (PA) of *Bacillus anthracis *[13] and the C-terminal part of the alpha toxin of *Clostridium perfringens *[14]. In the case of CotB-TTFC, dot blot experiments showed that each recombinant spore exposed 1.5 × 10^3 ^chimeric molecules [15]. CotC has been used to display the C-terminal fragment of the tetanus toxin (TTFC) [5], the B subunit of the heat-labile toxin (LTB) of *Escherichia coli *[5] and a tegumental protein of *Clonorchis sinensis *[16]. The CotC-based display on the spore surface has been found to depend on the site of insertion of the heterologous part. A 5-fold increase in the efficiency of display was observed when TTFC was located at the N-terminal end of CotC rather than at its C-terminal end [15]. The ability of recombinant spores expressing antigen to induce strong specific immune responses has highlighted the spore as a novel and promising mucosal vaccine delivery system [17]. A third coat protein, CotG [18], has also been used to display heterologous enzymes [6,7].

Here we report the use of all three previously utilized coat proteins, CotB, CotC and CotG, to display UreA, a urease subunit of *Helicobacter acinonychis*, an animal pathogen closely related to *H. pylori *and recognized as a useful *in vivo *model to study *H. pylori *virulence mechanisms [19]. UreA of *H. pylori *has been shown to induce high levels of protection against a *H. pylori *challenge infection in vaccinated mice [17,20,21]. Clinical studies (phase I) based on the use of UreA of *H. pylori *as antigen have been performed [22-24] and the use of that antigen patented (OraVax Inc., Cambridge, MA, US). However, there is evidence that UreA from different Helicobacter species can induce protection against *H. pylori *infection [25,26].

## Results

### The UreA of *Helicobacter acinonychis*

*H. acinonychis *produces a urease subunit A highly homologous to that of *H. pylori*. The UreA subunits of the two species share 96% identity with only 8 different amino acid residues out of 238. The *ureA *gene of *H. acinonychis *was PCR amplified and cloned in *Escherichia coli *in frame with a 6xHis tag under the transcriptional control of an inducible *ara *promoter. The protein was over-expressed, purified on Ni-columns and used to raise polyclonal antibody in mice. The obtained anti-UreA serum recognized UreA of *H. pylori *(data not shown), suggesting that the *H. acinonychis *protein can be used as an antigen to develop a new spore-based vaccine against *H. pylori*. The anti-UreA antibody failed to react against proteins extracted from *Bacillus subtilis *or *Escherichia coli *(data not shown), indicating that the recognition of UreA of *H. acinonychis *and *H. pylori *is specific and that the signal observed with *E. coli *expressing *ureA *of *H. acinonychis *is exclusively due to the heterologous protein.

### Construction and chromosomal integration of gene fusions

**Figure 1:**
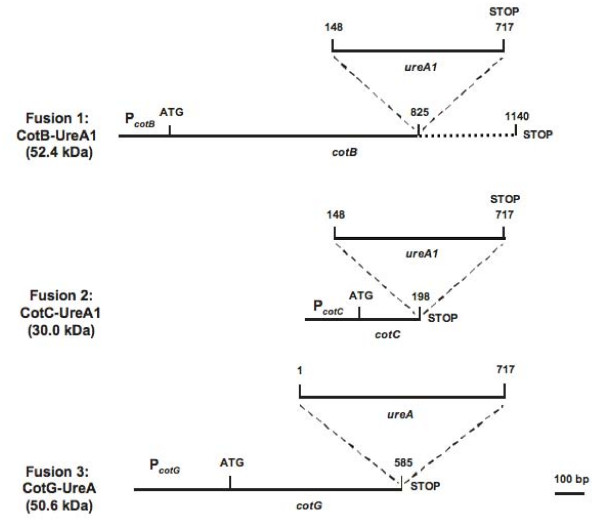
**Schematic representation of the three gene fusions obtained**. Either the entire coding region of the *ureA *gene (1-717) or part of it (148-717) was cloned in frame to nucleotide 825 of *cotB*, to nucleotide 198 of *cotC *and to nucleotide 585 of *cotG*. In Fusion 1 the dashed line represents the region of CotB not included in the chimeric protein (see text).

To obtain recombinant *B. subtilis *spores expressing UreA on their surface we used three *cot *proteins, CotB, CotC and CotG as carrier proteins. To this aim the coding part of the *ureA *gene of *H. acinonychis *was fused in frame with the coding part of *cotB*, *cotC *or *cotG*, as specified below. All gene fusions retained the promoter of the *cot *gene to ensure proper timing of expression during the sporulation process (Fig. 1). Genetic stability was obtained by integrating the gene fusions on the *B. subtilis *chromosome into the coding sequence of the non-essential gene *amyE *[27].

The C terminus of CotB is formed of three 27 amino acid repeats that confer genetic instability to chimeric proteins containing them [9]. For this reason, when CotB was used as a carrier, DNA encoding the three repeats was not included in the gene fusions and only DNA encoding the N-terminal 275 amino acid residues of CotB was used (Fig. 1). When CotC or CotG were used as carriers, DNA encoding the entire Cot proteins was used (Fig. 1).

As heterologous part we initially used the entire UreA subunit of 238 amino acids. However, while its fusion with CotG produced a stable chimeric protein (see below), with CotB and CotC we failed to observe any fusion product. For this reason we fused to CotB and CotC a shorter form of UreA, lacking 49 amino acids at its N-terminal end. With the shorter form of the antigen (*ureA1 *in Fig. 1), stable chimeric proteins were produced with CotB and CotC (see below).

Fusion 1 was obtained cloning *ureA1 *(570 bp) in frame with the serine codon at position 825 of *cotB*; Fusion 2 was constructed cloning *ureA1 *in frame with the tyrosine codon at position 198 of *cotC*; and Fusion 3 was obtained cloning a 717 bp DNA fragment containing the entire *ureA *gene in frame with the lysine codon at position 585 of *cotG *(Fig. 1).

All gene fusions were integrated on the *B. subtilis *chromosome and individual clones for each transformation, tested by PCR (not shown), were named KH17 (Fusion 1), KH10 (Fusion 2) and KH23 (Fusion 3) and used for further analysis.

The three recombinant strains and their isogenic parental strain PY79 showed comparable sporulation and germination efficiencies and their spores were equally resistant to chloroform and lysozyme treatment (not shown). Therefore, limited to the spore properties that we have analyzed, the presence of Cot-UreA fusions does not affect spore structure or functionality.

### Surface expression

To verify that the gene fusions were localized on the spore coat, we used a western blot approach using anti-CotB, anti-CotC, anti-CotG and anti-UreA antibodies. To perform the western blot experiments Fusion 2 was moved by chromosomal DNA-mediated transformations into isogenic *B. subtilis *strains deleted of *cotC *and/or *cotU *genes, since it has been previously reported that the presence of the wild type allele of *cotC *or of its homolog *cotU*, may reduce the efficiency of antigen display [4,5]. For the same reason Fusion 3 was moved into an isogenic strain deleted of *cotG *[18], while Fusion 1 was only analyzed in otherwise wild type cells, since it has been reported that the absence of a *cotB *wild type allele impairs display of the recombinant form of CotB [4].

**Figure 2:**
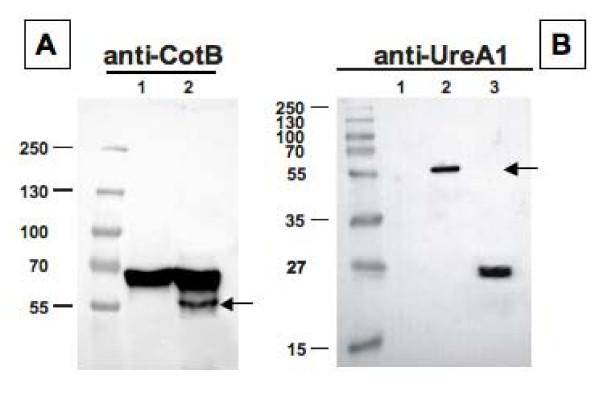
**Western blot analysis of Fusion 1 (CotB-UreA1) performed with anti-CotB (A) or anti-UreA (B) of spore coat proteins extracted from strains PY79 (lanes 1) and KH17 (PY79 carrying *cotB::ureA1*) (lanes 2)**. In both panels arrows point to fusion proteins. Purified UreA was run in lane 3 of panels B. Twenty five micrograms of total proteins were separated on either 8% (A), 12% (B) polyacrylamide gels, electrotransferred to nitrocellulose membranes and reacted with primary antibodies and then with peroxidase-conjugated secondary antibodies and visualized by the enhanced chemiluminescence method.

Western blot analysis of spore coat proteins purified from wild type and recombinant strains carrying Fusion 1 revealed the presence of an about 55-kDa band which reacted with both UreA- and CotB-specific antibodies (Fig. 2AB). A 66-kDa band, only reacting with CotB-specific antibody, was present in extracts from wild type and recombinant spores (Fig. 2AB), indicating the presence of intact CotB molecules in the spore coat together with CotB-UreA1 fusion protein.

**Figure 3:**
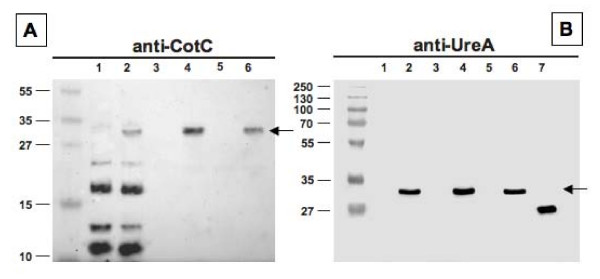
**Western blot analysis of Fusion 2 (CotC-UreA1) performed with anti-CotC (A) or anti-UreA (B) of spore coat proteins extracted from strains PY79 (lanes 1), KH10 (PY79 carrying *cotC::ureA1*) (lanes 2), RH101 (PY79 *cotC::spc*) (lanes 3), KH11 (PY79 *cotC::spc cotC::ureA1*) (lanes 4), RH209 (PY79 *cotC::spc cotU::erm*) (lanes 5) and KH12 (PY79 *cotC::spc cotU::erm cotC::ureA1*) (lanes 6)**. In both panels arrows point to fusion proteins. Purified UreA was run in lane 7 of panels B. Twenty five micrograms of total proteins were separated on either 15% (A) or 12% (B) polyacrylamide gels, electrotransferred to nitrocellulose membranes and reacted with primary antibodies and then with peroxidase-conjugated secondary antibodies and visualized by the enhanced chemiluminescence method.

The analysis of strains carrying Fusion 2 showed the presence of an about 30-kDa band which reacted with both UreA- and CotC-specific antibodies (Fig. 3AB). A standard pattern of CotC and CotU proteins [11,12] was observed in wild type spore with and without Fusion 2 (Fig. 3A, lanes 1-2). In agreement with a previous report [15], the fusion of a heterologous protein at the C terminus of CotC impaired the formation of CotC homodimer and CotC-CotU heterodimer. As a consequence, when fused to UreA1 CotC was only found as a monomer. Only the recombinant protein was observed in strains carrying either a null mutation in *cotC *and/or *cotU *(Fig. 3A, lanes 4-6).

**Figure 4:**
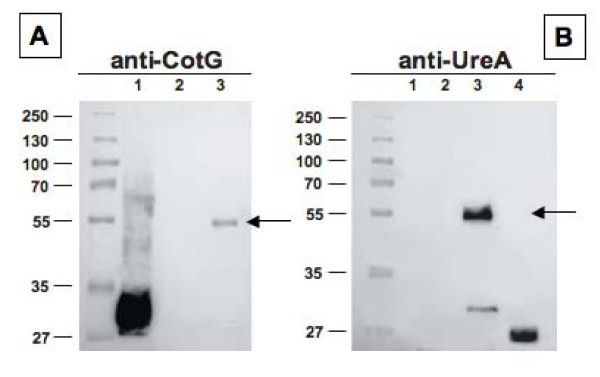
**Western blot analysis of Fusion 3 (CotG-UreA) performed with anti-CotG (A) or anti-UreA (B) of spore coat proteins extracted from strains PY79 (lanes 1), ER203 (PY79 *cotG::erm*) (lanes 2) and KH23 (PY79 *cotG::erm cotG::ureA*) (lanes 3)**. In both panels arrows point to fusion proteins. Purified UreA was run in lane 4 of panel F. Twenty five micrograms of total proteins were separated on either 15% (A) or 12% (B) polyacrylamide gels, electrotransferred to nitrocellulose membranes and reacted with primary antibodies and then with peroxidase-conjugated secondary antibodies and visualized by the enhanced chemiluminescence method.

The analysis of strains carrying Fusion 3 showed that in the presence of a wild type cotG allele the chimeric protein was not expressed (data not shown). However, the analysis of the strain carrying the gene fusion and a deletion of the wild type *cotG *gene, showed the appearance of a 55-kDa band able to react with both UreA- and CotG-specific antibodies (Fig. 4AB). A 32-kDa band, corresponding to CotG [24], was observed in wild type spores (Fig. 4A), while a band of about 30 kDa only reacting with anti-UreA antibody was present in coat extracts of a strain carrying a deletion of the *cotG *gene and Fusion 3 (Fig. 4B, lane 3). This band is not (or very weakly) recognized by anti-CotG antibody and is bigger that purified UreA (Fig. 4B, lane 4), we therefore hypothesize that it is a degradation product of CotG-UreA and contains only a small fragment of CotG.

In all three cases the recombinant proteins observed showed apparent molecular weights that correlated well with the deduced molecular weights: Fusion 1, 52.4/55; Fusion 2, 30/30; Fusion 3, 50.6/55 (deduced/apparent kDa).

### Surface display

**Figure 5:**
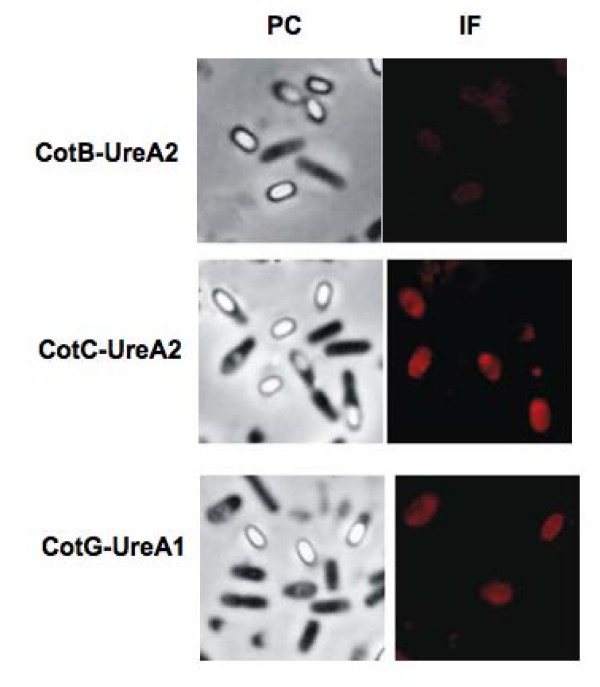
**Immunofluorescence microscopy analysis**. Sporulating cells and free spores are visualized by phase contrast (PC) and by immunofluorescence (IF) microscopy. Samples were labeled with mouse anti-UreA antisera, followed by anti-mouse IgG-TR (Texas Red or sulphorhodamine 101 acid chloride) conjugates. The same exposure time was used for all IF samples.

To analyse the surface exposure of Cot-fused UreA molecules, sporulating cells of wild type and the isogenic recombinant strains were analyzed by immunofluorescence microscopy with UreA-specific primary antibodies and anti-mouse IgG-TR (Texas Red) (Santa Cruz Biotechnology Inc.) as secondary antibody. While for Fusion 1 (CotB-UreA1) a weak fluorescence signal was observed around free, mature, spores (Fig. 5), for Fusion 2 (CotC-UreA1) and Fusion 3 (CotG-UreA) fluorescence was observed around forming spores (still inside mother cells) but not around free spores (Fig. 5). These results indicate that in the case of CotB-UreA1 the spore-exposed antigen is accessible to the antibody, while on spores carrying CotC- or CotG-UreA the antigen is present (since it can be extracted and visualized by western blot) but not accessible to the interaction with the antibody.

### Efficiency of expression

**Figure 6:**
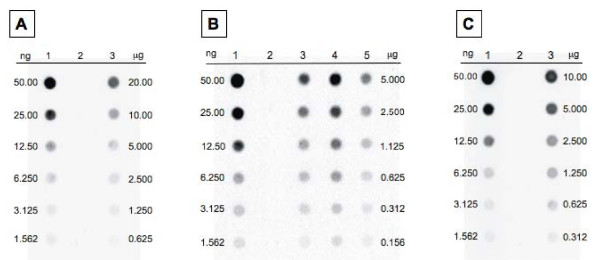
**Dot blot performed with the indicated concentrations of coat proteins (in μg) extracted from spores carrying Fusion 1 (A, lane 3), Fusion 2 (B, lanes 3-5) and Fusion 3 (C, lane 3) and from wild type spores (lane 2 in all panels)**. In panel B, lanes 3, 4 and 5 contain proteins of strains KH10 (PY79 carrying *cotC::ureA1*), KH11 (PY79 *cotC::spc cotC::ureA1*) and KH12 (PY79 *cotC::spc cotU::erm cotC::ureA1*), respectively. Purified UreA (in ng, lane 1 in all panels) was also utilized.

A quantitative determination of the amount of UreA present on *B. subtilis *spores was obtained by dot blot experiments using serial dilutions of purified UreA and of coat proteins extracted from spores of the wild type and the recombinant strains. Proteins were reacted with anti-UreA antibody, then with alkaline phosphatase-conjugated secondary antibodies and colour developed by the BCIP/NBT system (Bio-Rad) (Fig. 6). A densitometric analysis indicated that the CotB-UreA1 fusion protein amounted to 0.1% of total coat proteins extracted, CotC-UreA1 between 0.4 and 0.8%, depending on the genetic background utilized, and CotG-UreA to 0.5% (Table 1). Considering the average amount of total proteins extracted in our experimental conditions from each spore [4.6 mg/ml (± 0.23) for strain KH17, 4.9 mg/ml (± 0. 26) for strain KH10, 5.1 mg/ml (± 0.18) for strain KH11, 5.1 mg/ml (± 0.25) for strain KH12 and 4.7 mg/ml (± 0.31) for strain KH23], we calculated that the number of recombinant proteins extracted from each spore ranged between 1.1 × 10^3 ^(KH17) and 15 × 10^3 ^(KH11) (Table 1).

**Table 1:** Densitometric analysisa^a^

UreA source	Amount of proteins used (ng)	Density in OD/mm^2 ^(standard deviation)	UreA concentration (ng) in extracts (% of total)	n° of recombinant proteins extracted from each spore
**Fusion 1**				

Purified UreA	6.25 ng	38.9 (± 0.08)	NA	
	3.12 ng	19,1 (± 0.11)	NA	
	1.56 ng	9.8 (± 0.06)	NA	

KH17 (CotB-UreA1)	5.00 μg	30,4 (± 0,03)	4.93 (0.10)	
	2.50 μg	15,1 (± 0,01)	2.51 (0.10)	**1.1 × 10^3^**
	1.25 μg	7,4 (± 0,02)	1.12 (0.09)	

**Fusion 2**				

Purified UreA	25.0 ng	127.8 (± 0.02)	NA	
	12.5 ng	63,3 (± 0.01)	NA	
	6.25 ng	32.1 (± 0.04)	NA	

KH10 (CotC-UreA1 wt background)	2.50 μg	99.1 (± 0.07)	16.55 (0.66)	
	1.25 μg	48.6 (± 0.09)	8.14 (0.65)	**9.6 × 10^3^**
	0.625 μg	23.6 (± 0.10)	4.37 (0.70)	

KH11 (CotC-UreA1 *cotC *background)	2.50 μg	117.4 (± 0.03)	20.82 (0.83)	
	1.25 μg	57.7 (± 0.08)	10.35 (0.83)	**15 × 10**^3^
	0.625 μg	28.9 (± 0.21)	5.15 (0.82)	

KH12 (CotC-UreA1 *cotC cotU *background)	2.50 μg	63.9 (± 0.03)	11.10 (0.44)	
	1.25 μg	30.7 (± 0.01)	5.71 (0.46)	**7.2 × 10**^3^
	0.625 μg	15.4 (± 0.03)	2.93 (0.47)	

**Fusion 3**				

Purified UreA	25.0 ng	131.2 (± 0.30)	NA	
	12.5 ng	64,1 (± 0.11)	NA	
	6.25 ng	31.6 (± 0.22)	NA	

KH23 (CotG-UreA *cotG *background)	5.00 μg	135,7 (± 0.25)	28.42 (0.57)	
	2.50 μg	68,2 (± 0.37)	14.73 (0.59)	**5.3 × 10**^3^
	1.25 μg	34,7 (± 0,05)	7.15 (0.57)	

## Discussion

The A subunit of the urease (UreA) of various species of the *Helicobacter *genus, has long been recognized as an antigen able to induce high levels of protection against the infection by the human pathogen *H. pylori *[17,20,21,25,26]. The use of spores as mucosal vaccine vehicles has been tested with various antigens [4,5,13,14,16] and recently reviewed [28]. Expression of UreA on the spore was achieved by using three different spore coat proteins as carriers. All three proteins, CotB, CotC and CotG, were previously used to express and/or display heterologous proteins on the *B. subtilis *spore surface. In particular, CotB and CotC were used to express heterologous antigens [4,5,13,14,16], while CotG was used to express heterologous enzymes [6,7]. Our initial attempts of using the entire UreA subunit of 239 amino acid residues were not successful with CotB and CotC as carriers. However, we successfully used a shorter version of UreA, indicated here as UreA1 and lacking 49 amino acids at the N-terminal end of the antigen, with both CotB and CotC. UreA1, contains all six potentially most immunogenic regions of UreA that are all included between residue 64 and residue 189, as determined by analysis of the UreA protein sequence by the Antigen program (a part of EMBOSS package).

A first conclusion of this study comes from the analysis of the strain carrying the *cotB::ureA1 *gene fusion. Immunofluorescence experiments showed that, when fused to CotB, UreA1 is displayed on the spore surface, and dot-blot data followed by densitometric analysis indicated that 1.1 × 10^3 ^CotB-UreA1 molecules were extracted from each purified spore. A previous study, in which CotB was used to express the C fragment of the tetanus toxin (TTFC), showed that the heterologous part was displayed on the spore surface and that 1.5 × 10^3 ^recombinant proteins were extracted from each spore [4]. The similarities observed between spores carrying CotB-UreA1 and CotB-TTFC suggest that surface display and amount of recombinant protein expressed depends mainly on CotB and is not influenced by the nature of the heterologous part.

The functionality of CotC as a carrier appears, instead, to be dependent on the heterologous protein. When fused to CotC, UreA1 is efficiently expressed and is present on the coat but is not displayed on the spore surface. Two other antigens, TTFC and the B subunit of the heat-labile toxin of *E. coli *(LTB) were previously reported as surface displayed when fused to CotC [5]. However, the number of recombinant molecules extracted from each purified spore is reproducibly higher with CotC than with other two coat proteins as carriers and ranges from 7.2 × 10^3 ^and 15 × 10^3 ^depending on the genetic background of the host cell (Table 1).

Of the three coat proteins tested, CotG is the only one that allowed the expression of the entire UreA protein. UreA is not displayed but is present in the coat and is extracted in amounts about intermediate between those observed with the other two coat proteins (Table 1). However, the recombinant CotG-UreA protein is partially processed. While most CotG-UreA molecules have a size of about 55 kDa, that correlates well with the deduced size of 50.6 kDa, a fraction of these molecules is probably processed to originate a protein of about 30 kDa. This protein is smaller than UreA alone (Fig. 4B), is recognized by anti-UreA antibody and not recognized, or very poorly recognized by anti-CotG antidody (Fig. 4AB). We speculate that either the CotG-UreA chimera is unstable and partially degraded or that a proteolytic cleavage occurs within the CotG part of the chimera. In this case the 30 kDa protein would be formed by UreA and by a small fragment of CotG, explaining the size and the weak, if any, reactivity with anti-CotG antibody. However, a proteolytic processing has never been reported for wild type CotG. Additional experiments will be needed to explain the observed phenomenon and identify the presumptive cleavage site.

It is interesting to note that, for the various coat proteins used as carriers, the genetic background of the host cell differently affects the surface expression of the fusion proteins. CotC-based chimeras are better expressed in the absence of a wild type allele of *cotC *but in the presence of a wild type allele of *cotU*, coding for a CotC homolog, known to interact with CotC [12], while CotB-UreA1 is only expressed in the presence of a wild type allele of *cotB *and, on the contrary, CotG-UreA is only expressed in the absence of a wild type allele of *cotG*.

Results reported here point to CotB and CotC as the most appropriate carriers for UreA display on the spore surface and for its efficient expression, respectively. Immunological experiments will now be needed to assess whether the surface display of an antigen is an essential requisite for inducing a protective immune response or whether it is preferable to have the highest possible number of recombinant molecules on the spore coat layers even though these are not exposed on the spore surface. An antigen that is not directly exposed on the spore surface but is very abundant in the underneath protein layers, could be protected from the gastric enzymes and result immunologically more active.

## Conclusions

1) UreA of *H. acinonychis *was expressed on the spore of *B. subtilis*, a new heterologous expression system recently utilized to display antigens and enzymes [28]. Three different spore surface proteins were used as carrier to express UreA: CotB, CotC or CotG.

2) Among the three carriers, CotC was shown to allow the highest efficiency of expression. A large amount of its passenger protein was found to be located within the coat, however it was not displayed outside the spore. On the contrary, the level of expression of CotB-fused UreA was lower, but in this case the passenger protein was exposed on the spore surface and thus CotB resulted as a more appropriate carrier for the display of heterologous proteins. Finally CotG gave results similar to those with CotC, but the CotG-UreA recombinant protein appeared to be partially processed.

## Methods

### Bacterial strains and transformation

*Bacillus subtilis *strains used in this study are listed in Table 2. Plasmid amplification for nucleotide sequencing and subcloning experiments were performed with *Escherichia coli *strain DH5α [29]. Bacterial strains were transformed by previously described procedures: CaCl_2_-mediated transformation of *E. coli *competent cells [29] and transformation of *B. subtilis *[30].

**Table 2:** Strain list

Strain	Relevant genotype	Source
PY79	prototrophic	[34]

KH17	*amyE::cotB::ureA1*	This work

RH101	*cotC::spc*	[11]

RH209	*cotC::spc cotU::neo*	[15]

KH10	*amyE::cotC::ureA1*	This work

KH11	*cotC::spc amyE::cotC::ureA1*	This work

KH12	*cotC::spc cotU::neo amyE::cotC::ureA1*	This work

ER203	*cotG::erm*	[18]

KH23	*amyE::cotG::ureA cotG::erm:*	This work

### Construction of gene fusions

To obtain various gene fusions DNA coding for the selected coat protein was PCR amplified using the *B. subtilis *chromosome as a template and as primers oligonucleotide pairs cotB-up/cotB-dn, cotC-up/cotC-dn and cotG-up and cotG-dn (Table 3) for fusions *cotB::ureA1, cotC::ureA1 *and *cotG-ureA*, respectively. Amplification products of 1100 bp (cotB::ureA1), 393 bp (*cotC::ureA1*) and 1043 bp (*cotG::ureA*) were obtained and cloned into the pGem-T easy vector (Promega) or pDL vector (*Bacillus *Genetic Stock Center) for *cotG::ureA*, yielding plasmids pGEM-CotB, pGEM-CotC and pDL-CotG.

**Table 3:** Oligonucleotides list

Name	Sequence (5'-3')	Restriction site
cotB-up	**AAGCTT**ACGGATTAGGCCGTTTGTC	*Hind*III

cotB-dn	**GATATC**GGATGATTGATCATCTGAAG	*Eco*RV

cotC-up	ACCC**AAGCTT**TGTAGGATAAATCGTTTG	*Hind*III

cotC-dn	**GATATC**GTAGTGTTTTTTATGCTT	*Eco*RV

cotG-up	CCC**GAATTC**CGAGAAAAAATCC	*Eco*RI

cotG-dn	CTT**GGATCC**TTTGTATTTCTTTTTGACTAC	*Bam*HI

ureA-up	GAG**GGATCC**ATGAAACTCACCCCAAAAG	*Bam*HI

ureA-dn	CGC**GAGCTC**TAGGGCCATACATAGAAAC	*Sac*I

ureA1-up	GAAGCG**GATATC**GGTAAAAAGACTGCG	*Eco*RV

ureA1-dn	GGGCCATAC**ACTAGT**ACATATTCTTTTCTGCTAATC	*Spe*I

hisureA-up	CTC**GGTACC**TTCTTTTCTGCTAATCTTTTTTTC	*Kpn*I

hisureA-dn	TAT**GCTAG**CATGCATCATCACCATCACCATCACTCGAAACTCACCCCAAAAGAG	*Nhe*I

In bold are the recognition sites for the restriction enzymes indicated in the table.

A 625 bp DNA fragment coding for a fragment of UreA was PCR amplified using *H. acinonychis *chromosome as a template and oligonucleotides ureA1-up and ureA1-dn (Table 3) as primers. The PCR product was sequentially digested with *EcoRV *and *Spe*I and cloned in frame to the 3' end of the *cotB *and *cotC *genes carried by plasmids pGEM-CotB and pGEM-CotC, yielding plasmids pKH09 and pKH02, respectively. Plasmids pKH09 and pKH02 were digested with *Hind*III and *EcoR*I and fragments carrying the gene fusions gel-purified and ligated into plasmid pDG364 [27] previously digested with the same two restriction enzymes, yielding plasmids pKH14 and pKH03, respectively. A 748 bp DNA fragment coding for the entire UreA subunit was PCR amplified using *H. acinonychis *chromosome as a template and oligonucleotides ureA-up and ureA-dn as primers (Table 3). The PCR product was sequentially digested with *Bam*HI and *Sac*I and cloned in frame to the 3' end of the *cotG *gene carried by plasmid pDL-CotG yielding plasmid pKH20.

### Chromosomal integration

Plasmids pKH14 and pKH03 were linearized by digestion with *XhoI *while plasmid pKH20 was linearized by digestion with *PstI*. Linearized DNA was used to transform competent cells of the *B. subtilis *strain PY79. Chloramphenicol-resistant (Cm^R^) clones were the result of a double-crossover recombination event, resulting in the interruption of the non-essential *amyE *gene on the *B. subtilis *chromosome. Several Cm^R ^clones were tested by PCR using chromosomal DNA as a template and oligonucleotides AmyS and AmyA [9] to prime the reaction. Three clones, one for each transformation, were selected, called KH17 (from pKH14, Fusion 1), KH10 (from pKH03, Fusion 2), and KH21 (from pKH20, Fusion 3) and kept for further studies.

Chromosomal DNA extracted from strain KH10 was moved into a isogenic *cotC *null strain RH101 [5], and isogenic *cotC cotU *double null strain RH209 [15] by chromosomal DNA-mediated transformation [27] yielding strains KH11 and KH12, respectively. Chromosomal DNA extracted from strain KH21 was used to transform the isogenic cotG null strain ER203 [18], yielding strains KH23.

### Preparation of spores

Sporulation was induced by the exhaustion method in DS (Difco-Sporulation) medium as described elsewhere [31]. Sporulating cultures were harvested 24 h after the initiation of sporulation and purified using a lysozyme treatment to break residual sporangial cell followed by washing steps in 1 M NaCl, 1 M KCl and water (two-times), as described previously [31]. PMSF (0.05 M) was included to inhibit proteolysis. After the final suspension in water, spores were treated at 65°C for 1 h to kill residual cell. The spore suspension was titrated immediately for CFU/ml before freezing at -20°C. By this method we could reliably produce 6 × 10^10 ^spores per liter of DSM culture. Sporulation and germination efficiencies were measured as previously reported [27]. Alanine and asparagine were used to induce germination.

### Extraction of spore coat proteins

Spore coat proteins were extracted from 50 μl of a suspensions of spores at high density (1 × 10^10 ^spores per ml) using a Decoating extraction buffer as described elsewhere [32]. Extracted proteins were assessed for integrity by SDS-polyacrylamide gel electrophoresis (PAGE) and for concentration by two independent methods: the Pierce BCA Protein Assay (Pierce) and the BioRad DC Protein Assay kit (Bio-Rad).

### Western and dot blot analyses

Extracted proteins were separated in 8%, 12% or 15% denaturing polyacrylamide gels, electrotransferred to nitrocellulose filters (PerkinElmer) and used for Western blot analysis by standard procedures. Western blot filters were visualized by the enhanced chemiluminescence (PerkinElmer) method as specified by the manufacturer. Serial dilutions of extracted proteins and of purified UreA were used for dot blot analysis. The filters were then visualized by the enhanced chemiluminescence (PerkinElmer) method and subjected to densitometric analysis with Chemidoc XRS (Bio-Rad) and the MultiAnalyst software.

### Immunofluorescence microscopy

*B. subtilis *strains (PY79, KH17, KH10 and KH23) were induced to sporulate by the exhaustion method [27]. Samples were collected at different times after the onset of sporulation and fixed directly in the medium as described by Harry et al., [33], with the following modifications: after suspension in GTE-lysozyme (50 mM glucose, 20 mM Tris- HCl [pH 7.5], 10 mM EDTA, 2 mg of lysozyme/ml), samples (30 μl) were immediately applied to microscope slide previously coated with 0.01% (wt/vol) poly-L-lysine (Sigma). After 3 min, the liquid was removed and the microscope slide allowed to dry (2 h at room temperature). The microscope slides were washed three times in phosphate-buffered saline (PBS) (pH 7.4), blocked for 30 min with 3% milk in PBS at room temperature and then washed nine more times with PBS. Samples were incubated overnight at 4°C with anti-UreA antibody (raised in mouse), washed ten times, and then incubated with anti-mouse IgG-TR conjugates with Texas Red or sulphorhodamine 101 acid chloride (Santa Cruz Biotechnology, Inc.) for 2 h at room temperature. After ten washes the coverslip was mounted onto a microscope slide and viewed using an Olympus BX51 fluorescence microscope using the same exposure time for all samples. Images were captured using a Olympus DP70 digital camera, processed with analySIS software and saved in TIFF format.

### Purification of UreA and antibody production

The *ureA *gene of *H. acinonichis *was PCR amplified using chromosomal DNA as a template and oligonucleotides hisureA-up and hisureA-dn (Table 3) as primes. DNA encoding six histidines (His6-tag) was carried by oligonucleotide hisureA-dn. The obtained PCR product of 737 bp was digested with enzymes *Kpn*I and *Nhe*I and cloned into the commercial vector pBAD (Stratagene). The resulting plasmid, pMD1, was verified by restriction analysis and nucleotide sequencing. pMD1 was used to transform the *E. coli *strain DH5α and the recombinant strain used to overproduce UreA by addition of arabinose 0.05%. A 27 kDa protein was visualized on a blue-coomassie stained gel and purified on Ni-NTA superflow agarose (Qiagene) followed by gel filtration on Superose 6 resin. 0.7 mg of pure UreA protein were obtained from 3 liters of culture. For antibody production six C57BL/6J mice were immunised intraperitoneally with 30 μg of purified UreA per mouse with incomplete Freund's adjuvant in a total volume of 300 μl. The injections took place at day 0, 14, 35 and 56. At day 24 and 45 sera samples were taken by tail bleeding. At day 66 total blood was collected. Obtained sera were tested against purified protein and optimal dilution of anti-UreA sera was established as 1:100 000 for western blot analysis.

## Competing interests

The authors declare that they have no competing interests.

## Authors' contributions

KH - performed most of the experiments; RI - contributed to the immunofluorescence experiment and to the analysis of dot blot experiments; MD - purified UreA and analyzed it by western blotting; JK - cloned the *ureA *gene of *H. acinonychis*; AI - contributed to manuscript writing; GPS - prepared the anti-UreA antibody in mice; MDF - contributed to experiment design and manuscript writing; MO - contributed to experiment design and manuscript writing; ER - contributed discussions and suggestions during the work and wrote most of the manuscript. All authors read and approved the final manuscript.
